# Accuracy of 18-F Fluorodeoxyglucose Positron Emission Tomographic/Computed Tomographic Imaging in Primary Staging of Squamous Cell Carcinoma of the Oral Cavity

**DOI:** 10.1001/jamanetworkopen.2021.7083

**Published:** 2021-04-21

**Authors:** Christian Linz, Roman C. Brands, Theresia Herterich, Stefan Hartmann, Urs Müller-Richter, Alexander C. Kübler, Lukas Haug, Olivia Kertels, Thorsten A. Bley, Alexander Dierks, Andreas K. Buck, Constantin Lapa, Joachim Brumberg

**Affiliations:** 1 Department of Oral and Maxillofacial Plastic Surgery, University Hospital of Würzburg, Würzburg, Germany; 2 Comprehensive Cancer Center Mainfranken, University Hospital of Würzburg, Würzburg, Germany; 3 Department of Pathology, University of Würzburg, Würzburg, Germany; 4 Department of Diagnostic and Interventional Radiology, University Hospital of Würzburg, Würzburg, Germany; 5 Department of Nuclear Medicine, University Hospital of Würzburg, Würzburg, Germany; 6 Nuclear Medicine, Medical Faculty University of Augsburg, Augsburg, Germany

## Abstract

**Importance:**

Squamous cell carcinoma (SCC) of the oral cavity is one of the most common tumor entities worldwide. Precise initial staging is necessary to determine a diagnosis, treatment, and prognosis.

**Objective:**

To examine the diagnostic accuracy of preoperative 18-F fluorodeoxyglucose (FDG) positron emission tomographic/computed tomographic (PET/CT) imaging in detecting cervical lymph node metastases.

**Design, Setting, and Participants:**

This prospective diagnostic study was performed at a single tertiary reference center between June 1, 2013, and January 31, 2016. Data were analyzed from April 7, 2018, through May 31, 2019. Observers of the FDG PET/CT imaging were blinded to patients’ tumor stage. A total of 150 treatment-naive patients with clinical suspicion of SCC of the oral cavity were enrolled.

**Exposures:**

All patients underwent FDG PET/CT imaging before local tumor resection with selective or complete neck dissection.

**Main Outcomes and Measures:**

The accuracy of FDG PET/CT in localizing primary tumor, lymph node, and distant metastases was tested. Histopathologic characteristics of the tissue samples served as the standard of reference.

**Results:**

Of the 150 patients enrolled, 135 patients (74 [54.8%] men) with a median age of 63 years (range, 23-88 years) met the inclusion criteria (histopathologically confirmed primary SCC of the oral cavity/level-based histopathologic assessment of the resected lymph nodes). Thirty-six patients (26.7%) in the study cohort had neck metastases. Use of FDG PET/CT detected cervical lymph node metastasis with 83.3% sensitivity (95% CI, 71.2%-95.5%) and 84.8% specificity (95% CI, 77.8%-91.9%) and had a negative predictive value of 93.3% (95% CI, 88.2%-98.5%). The specificity was higher than for contrast-enhanced cervical CT imaging (67.0%; 95% CI, 57.4%-76.7%; *P* < .01) and cervical magnetic resonance imaging (62.6%; 95% CI, 52.7%-72.6%; *P* < .001). Ipsilateral lymph node metastasis in left- or right-sided primary tumor sites was detected with 78.6% sensitivity (95% CI, 63.4%-93.8%) and 83.1% specificity (95% CI, 75.1%-91.2%), and contralateral metastatic involvement was detected with 66.7% sensitivity (95% CI, 28.9%-100.0%) and 98.6% specificity (95% CI, 95.9%-100.0%). No distant metastases were observed.

**Conclusions and Relevance:**

In this study, FDG PET/CT imaging had a high negative predictive value in detecting cervical lymph node metastasis in patients with newly diagnosed, treatment-naive SCC of the oral cavity. Routine clinical use of FDG PET/CT might lead to a substantial reduction of treatment-related morbidity in most patients.

## Key Points

**Question:**

What is the diagnostic accuracy of 18-F fluorodeoxyglucose positron emission tomographic/computed tomographic imaging in squamous cell carcinoma of the oral cavity for detection of metastases?

**Findings:**

This diagnostic study including 135 patients showed 83.3% sensitivity, 84.8% specificity, and a negative predictive value of 93.3% with use of 18-F fluorodeoxyglucose positron emission tomographic/computed tomographic imaging for detecting cervical lymph node metastases. The specificity was significantly higher than observed for use of cervical computed tomography and magnetic resonance imaging.

**Meaning:**

Owing to its high negative predictive value, preoperative 18-F fluorodeoxyglucose positron emission tomographic/computed tomographic imaging may be useful to prevent overtreatment in more than 70% of patients with category T1 to T4 tumors of the oral cavity.

## Teaser

This diagnostic study examines the accuracy of F-18 fluorodeoxyglucose positron emission tomographic/computed tomographic imaging for detection of primary tumor, lymph node, and distant metastases in patients with oral cavity squamous cell carcinoma.

## Introduction

Cancer of the oral cavity and the oropharynx is the sixth most common tumor entity and the ninth most frequent cause of death worldwide.^1,2^ The annual estimated incidence of squamous cell carcinoma (SCC) of the oral cavity is approximately 275 000, and these cases represent more than 90% of all oral cancers.^3,4^ However, the diagnosis is determined late in up to 50% of patients and thus the condition is associated with a poor prognosis; the survival rate is approximately 60%, and the estimated recurrence rate is approximately 30% at 5-year follow-up.^5,6^ The presence of cervical lymph node metastasis is one of the most important adverse prognostic factors.^7,8,9,10^ Distant metastases, although rare, are generally considered incurable and therefore alter the therapeutic regimen.^11^

Precise initial tumor staging is necessary to determine a diagnosis, treatment, and prognosis. This staging requires accurate detection of (1) the primary tumor (T), (2) cervical lymph nodal involvement (N), and (3) distant metastases (M).^12,13^ In addition to a clinical examination, established imaging techniques for primary staging include ultrasonography, computed tomography (CT), and magnetic resonance imaging (MRI).^14,15^ Despite improvements in imaging, the overall rate of occult (clinically and radiologically undetectable) metastases in SCC of the oral cavity is approximately 15% to 34%.^1,16,17,18,19^ Selective neck dissection thus maximizes survival probability but leads to overtreatment, which is accompanied by reduced quality of life in approximately 75% of patients.^20^ Preoperative whole-body screening methods with a reliable prediction of N0 status of necks might help to reduce this rate. The value of positron emission tomography (PET) with 18-F fluorodeoxyglucose (FDG) in the preoperative staging of head and neck SCC has been demonstrated in the literature.^21,22,23,24,25,26^ However, only a few prospective studies that used FDG PET/CT imaging in cohorts with SCC of the oral cavity have thus far been conducted.^24,27^ A 2019 multicenter study obtained a high negative predictive value of FDG PET/CT in clinically noted N0 status of the neck sides of patients with head and neck SCC after imaging via MRI and/or CT.^28^ However, the preselection used in the study impedes general conclusions as to the diagnostic accuracy of FDG PET/CT and does not reveal whether this procedure can replace stand-alone morphologic imaging.

The aim of the present study was therefore to prospectively examine the diagnostic accuracy of FDG PET/CT in detecting cervical lymph node metastasis in patients with newly diagnosed SCC of the oral cavity who are treatment-naive. Particular importance was given to the negative predictive value of FDG PET/CT and to detecting lymph node metastases based on their localization ipsilateral or contralateral to the primary tumor site. We furthermore compared the diagnostic measures of FDG PET/CT with those of both cervical MRI and contrast-enhanced CT in the study cohort. In addition, we report on the detection rate of FDG PET/CT with regard to second primary tumors.

## Methods

### Study Design and Patients

From June 1, 2013, to January 31, 2016, a total of 150 patients with clinical suspicion of SCC of the oral mucosa were prospectively enrolled. Whole-body FDG PET/CT and cervical MRI were performed before further invasive interventions (panendoscopy and/or acquisition of biopsy samples). All patients underwent surgery within 2 weeks of the imaging workup. Resected primary tumors and lymph nodes were histopathologically evaluated. Data were analyzed from April 7, 2018, through May 31, 2019. The institutional review board of the University Hospital of Würzburg, Würzburg, Germany, approved this study, and written, informed consent was obtained from all participants. This study followed the Standards for Reporting of Diagnostic Accuracy (STARD) reporting guideline for diagnostic studies.^29^

The inclusion criteria for this study were defined as no previous treatment, histopathologically confirmed primary SCC of the oral cavity, and a level-based histopathologic assessment of resected lymph nodes. Patients with (1) tumor relapse, (2) cancer of unknown primary site, (3) en bloc resection of lymph node levels, or (4) uncertainties regarding the removed lymph node levels were excluded.

### Imaging

Imaging for all patients was performed on an integrated PET/CT scanner (Siemens Biograph mCT 64; Siemens Healthineers). Before imaging, patients fasted for at least 4 to 6 hours. Blood glucose levels were measured and confirmed to be below 160 mg/dL (to convert to millimoles per liter, multiply by 0.0555) before the intravenous injection of FDG, 300 ± 25 megabecquerels. After a distribution period of 60 minutes, PET emission data were acquired in 3-dimensional mode with a 200 × 200 matrix with 2-minute emission time per bed position from the vertex of the skull to the proximal thighs. Consecutively, transmission data were acquired using contrast-enhanced spiral CT (dose modulation with a quality reference of 180 mA per second, 120 kV, a 512 × 512 matrix, 5-mm slice thickness, an increment of 30 mm/s, a rotation time of 0.5 seconds, and a pitch index of 1.4). Furthermore, a dedicated acquisition of the head and neck with 1 bed position, a 3-minute emission time, and a contrast-enhanced CT was performed (180 mA per second, 120 kV, a 512 × 512 matrix, 3-mm slice thickness, an increment of 30 mm/s, a rotation time of 1.0 seconds, and a pitch index of 0.9). PET data were reconstructed iteratively (3 iterations, 24 subsets, a gaussian filtering of 2.0 mm full width at half maximum) with attenuation correction using dedicated standard software (HD-PET, Siemens Esoft, Siemens Healthineers).

Two experienced, board-certified nuclear medicine physicians (C.L. and J.B.) with access to relevant clinical data independently rated whole-body and cervical PET/CT imaging results (syngo.via workstation; Siemens Healthineers). The foci of increased tracer uptake with reference to healthy tissue and blood pool and/or the presence of morphologic alterations on CT images were recorded as being positive for tumor involvement. The localization, expansion, and infiltration of osseous structures, as well as the presence and number of nodal metastases, were recorded for each cervical lymph node level. Lymph node levels were assessed according to the imaging-based nodal classification.^30,31^ Furthermore, whole-body scans were evaluated for distant nodal and organ metastasis as well as for secondary cancer. Any initial difference in rating between the 2 readers was resolved via a subsequent consensus reading. If such a difference was present, the maximal and peak standardized uptake values (SUVs) of the primary tumor, the hottest cervical lymph node, and the hottest distant metastasis were measured.

### Surgery and Specimen Analysis

The operative care of the patients was carried out in line with institutional standards of care and valid guidelines.^19^ Surgical treatment consisted of resecting the local tumor and performing a selective neck dissection (levels I-III; levels I-III and Va; or levels II, III, and Va) or a complete neck dissection of levels I to V according to the Robbins classification.^32^ In the case of a negative FDG PET/CT result, we performed the recommended neck dissection without changing the initial surgical plan. In the case of positive FDG PET/CT findings on the contralateral side of the tumor, an additional neck dissection was performed.

Specimens were analyzed by a single experienced pathologist blinded to imaging results. The assessment was carried out with regard to tumor size, lymph node metastases, lymph vessel and venous invasion, perineural infiltration, resection status, and tumor grading according to the TNM classification of SCC of the oral cavity.^13^

### Statistical Analysis

We aimed to determine the sensitivity, specificity, positive predictive value (PPV), and negative predictive value (NPV) of FDG PET/CT with a CI of a maximum of ±15%.^33^ Based on previous publications on stand-alone FDG-PET^33^ and an expected dropout rate of 10%, we considered the enrollment of 150 patients to be sufficient.

Statistical analyses were performed using SPSS, version 24.0 (IBM Corp) and R, version 3.6.1 (R Foundation for Statistical Computing).^43^ We assessed sensitivity, specificity, PPV, and NPV of FDG PET/CT in detecting primary tumors, osseous infiltration, and the presence of cervical lymph node metastasis on histopathologic analysis. In addition, the diagnostic performance of FDG PET/CT was calculated for the nodal metastatic involvement of cervical sides and lymph node levels. In 125 patients of the study cohort, these measures were also compared with morphologic imaging via MRI and contrast-enhanced CT; detailed description of imaging procedures, image analysis, and statistical analysis are reported in the eMethods in the Supplement. Regarding FDG PET/CT, Spearman ρ correlational analysis was performed for the association between the uptake of the primary tumor (maximal and peak SUV) as well as for grading and the tumor size as measured in histopathologic examination. The maximal and peak SUV values of the primary tumor and the hottest lymph node in patients rated as positive in FDG PET/CT were furthermore compared via the Mann-Whitney test regarding the presence and absence of lymph node metastases.

To address the particular clinical relevance of detecting nodal involvement of the contralateral side, a subgroup analysis was performed in unilaterally located primary tumors. Because metastatic spread might differ in the tongue, this analysis was performed separately for primary tumors of the tongue and for tumors of all other primary sites. The diagnostic accuracy of side involvement was performed for centrally or bilaterally located tumors for both groups. Unilateral tumors (left or right) were assessed regarding the detection of nodal involvement of the ipsilateral and contralateral neck. Quantitative values are displayed as mean (SD) or as median and range, as appropriate. Using 2-sided, unpaired *t* test analysis, findings were considered significant at *P* < .05.

## Results

Of 150 patients, a total of 135 (74 [54.8%] men, 61 [45.2%] women) individuals with a median age of 63 years (range, 23-88 years) met the inclusion criteria and were enrolled in this study; 15 patients who had cancer other than SCC of the oral cavity were excluded (eFigure in the Supplement). Of the included participants, 114 patients (84.4%) had a primary tumor located on either the left (n = 60) or right (n = 54) side, 33 of whom had a unilateral primary tumor of the tongue. Twenty-one patients (15.6%) had a central or bilateral primary tumor (none with a tongue carcinoma). The primary tumor sites and localizations are listed in Table 1.

**Table 1.** Patient Characteristics

Characteristic	No. (%)
Age, mean (range), y	63 (23-88)
Sex	
Male	74 (54.8)
Female	61 (45.2)
Total	135 (100)
Localization of primary tumor	
Buccal mucosa	5 (3.7)
Central/lateral	0/5
Lips	7 (5.2)
Central/lateral	4/3
Mandibular mucosa	37 (27.4)
Central/lateral	3/34
Maxillary mucosa	16 (11.9)
Central/lateral	1/15
Floor of the mouth	37 (27.4)
Central/lateral	13/24
Tongue	33 (24.4)
Central/lateral	0/33
Total	135 (100)
Lymph node dissection	
I-III	91
I-III, Va	46
II, III, Va	35
I-V	57
Total	229
Primary tumor category	
pT1	59 (43.7)
pT2	41 (30.4)
pT3	4 (3.0)
pT4	31 (23.0)
Total	135 (100)
Local lymph node metastases	
pN0	99 (73.3)
pN1	18 (13.3)
pN2a	1 (0.7)
pN2b	14 (10.4)
pN2c	3 (2.2)
Total	135 (100)
Grading	
G1	21 (15.6)
G2	79 (58.5)
G3	27 (20.0)
G4	3 (2.2)
NA	5 (3.7)
Total	135 (100)

Abbreviation: NA, not available.

### Surgery

All 135 patients underwent primary tumor resection and neck dissection. In 2 cases, the surgeon decided to extend the lymph node dissection to the contralateral side owing to suspicious findings noted in the FDG PET/CT. Overall, 229 sides of lymph node dissections (left, right, and bilateral) were performed: 91 selective neck dissections with extirpation of levels I to III; 46 of levels I to III and Va; 35 of levels II, III, and Va; and 57 complete neck dissections (levels I-III, IV, and V). Levels I, II, and V were subdivided into a and b. In total, 1226 levels and sublevels were removed.

### Histopathologic Analysis and Imaging

Histopathologic analysis revealed a tumor category of T1 in 59 patients (43.7%) and T2 in 41 patients (30.4%). Four patients had category T3 (3.0%), and 31 had category T4 (23.0%). Tumor grading is displayed in Table 1. Ninety-nine patients (73.3%) had no lymph node metastasis (N0), and 36 patients (26.7%) had cervical lymph node metastasis, 18 of whom had a stage of N1 (13.3%) with 1 patient classified as N2a (0.7%), 14 as N2b (10.4%), and 3 as N2c (2.2%). Lymph node metastases were found in 6 patients (10.2%) with category T1, 16 patients (39.0%) with category T2, in 2 patients (50.0%) with category T3 tumors, and 12 patients (38.7%) with category T4 tumors. In total, 81 of the 5515 assessed lymph nodes (1.5%) contained metastases, which were located across 52 different neck levels (4.2% of 1226 assessed nodal levels). None of the patients investigated showed distant metastasis. Tissue samples for histopathologic analysis of 9 lesions that were suspicious for a secondary cancer in FDG PET/CT imaging were obtained in 8 patients. A solid second primary tumor was confirmed in 6 cases (1 of the glottis, thyroid, lung, and uterus, and 2 of the prostate); 2 patients showed precursor lesions (intraepithelial neoplasia of the colon) and 1 patient had a false-positive finding in the left tonsil.

In 132 of 135 patients (sensitivity, 97.8%; 95% CI, 95.3%-100.0%), the primary tumor was detected via FDG PET/CT (maximal SUV: 16.4 [range, 3.2-59.2]; peak SUV: 10.5 [range, 2.4-44.8]). For 31 patients with osseous infiltration, sensitivity was 87.1% (95% CI, 75.3%-98.9%) and specificity was 92.3% (95% CI, 87.2%-97.4%) (PPV: 77.1%; 95% CI, 63.2%-91.1%; NPV: 96.0%; 95% CI, 92.1%-99.8%) (Table 2 and Table 3). Correlational analysis revealed a significant association between tumor size and uptake (maximal SUV: ρ = 0.62, *P* < .001; peak SUV: ρ = 0.66; *P* < .001). The uptake of primary tumors in patients with cervical lymph node metastases was higher than in patients without nodal metastases (maximal SUV: 20.8 [range, 6.6.-47.8] vs 11.7 [range, 3.2-59.2]; *P* < .001; peak SUV: 13.1 [range, 4.8-28.4] vs 6.9 [range, 2.4-44.8]; *P* < .001). Likewise, the uptake in the hottest lymph node was higher in patients with cervical metastases than in patients without lymph node metastasis (maximal SUV: 10.8 [range, 3.6-29.5] vs 5.7 [range, 4.0-8.4]; *P* < .001; peak SUV: 6.5 [range, 2.2-24.8] vs 3.4 [range, 2.4-5.4]; *P* = .004).

**Table 2.** Estimated and True Conditions of FDG PET/CT for Oral Cavity Squamous Cell Carcinoma

Diagnostic question	No.				
	Total	True-positive	False-positive	False-negative	True-negative
Primary tumor	135	132	0	3	0
Osseous infiltration	135	27	8	4	96
Lymph node metastases					
Per patient	135	30	15	6	84
Per cervical sides	229	30	19	11	169
Per cervical lymph node level	1226	32	46	20	1128

Abbreviations: CT, computed tomography; FDG, 18-F fluorodeoxyglucose; PET, positron emission tomography.

**Table 3.** Diagnostic Accuracy of FDG PET/CT for Oral Cavity Squamous Cell Carcinoma

Diagnostic question	% (95% CI)				
	Prevalence	Sensitivity	Specificity	Predictive value	
				Positive	Negative
Primary tumor	100.0 (100.0-100.0)	97.8 (95.3-100.0)	NA	NA	NA
Osseous infiltration	23.0 (15.9-30.1)	87.1 (75.3-98.9)	92.3 (87.2-97.4)	77.1 (63.2-91.1)	96.0 (92.1-99.8)
Lymph node metastases					
Per patient	26.7 (19.2-34.1)	83.3 (71.2-95.5)	84.8 (77.8-91.9)	66.7 (52.9-80.4)	93.3 (88.2-98.5)
Per cervical sides	17.9 (12.9-22.9)	73.2 (59.6-86.7)	89.9 (85.6-94.2)	61.2 (47.6-74.9)	93.9 (90.4-97.4)
Per cervical lymph node levels	4.2 (3.1-5.4)	61.5 (48.3-74.8)	96.1 (95.0-97.2)	41.0 (30.1-51.9)	98.3 (97.5-99.0)

Abbreviations: CT, computed tomography; FDG, 18-F fluorodeoxyglucose; NA, not available; PET, positron emission tomography.

Thirty of 36 patients were correctly evaluated as having metastatic nodal involvement (sensitivity, 83.3%; 95% CI, 71.2%-95.5%; specificity: 84.8%; 95% CI, 77.8%-91.9%; PPV: 66.7%; 95% CI, 52.9%-80.4%; NPV: 93.3%; 95% CI, 88.2%-98.5%). The assessment of a cervical side as being either positive or negative for 1 or more lymph node metastases was calculated (sensitivity, 73.2%; 95% CI, 59.6%-86.7%; specificity, 89.9%; 95% CI, 85.6%-94.2%; PPV: 61.2%; 95% CI, 47.6%-74.9%; NPV: 93.9%; 95% CI, 90.4%-97.4%). The results of subgroup analysis regarding the primary tumor site are listed in eTable 1 and eTable 2 in the Supplement. A false-positive evaluation of cervical sides was more frequent for T4 tumors (32.2%) than for patients with T1 (11.9%) or T2 tumors (7.3%). In all but 1 patient with false-positive findings (Figure, A), histopathologic specimens contained ulcerating primary tumors and/or additional acute or chronic inflammatory findings (eg, mucosal inflammation, osteomyelitis, chronic lymphadenitis, and sarcoid-like lesions). The remaining patient displayed signs of acute tonsillitis on FDG PET/CT. False-negative events occurred in 3.4% of participants with category T1 tumors, in 14.6% of patients with category T2 tumors, in no patients with category T3 tumors, and in 9.7% of patients with category T4 tumors. The missed metastases in false-negatively scored individuals were micrometastases or were less than or equal to a mean size of 7 mm in diameter (mean [SD], 3.8 [1.7] mm). For the ipsilateral side, true-negative findings existed for patients with unilateral category T1 or T2 tumors in 93.8% of the cases. This negative predictive value decreased to 80.0% for patients with category T4 tumors.

**Figure.**
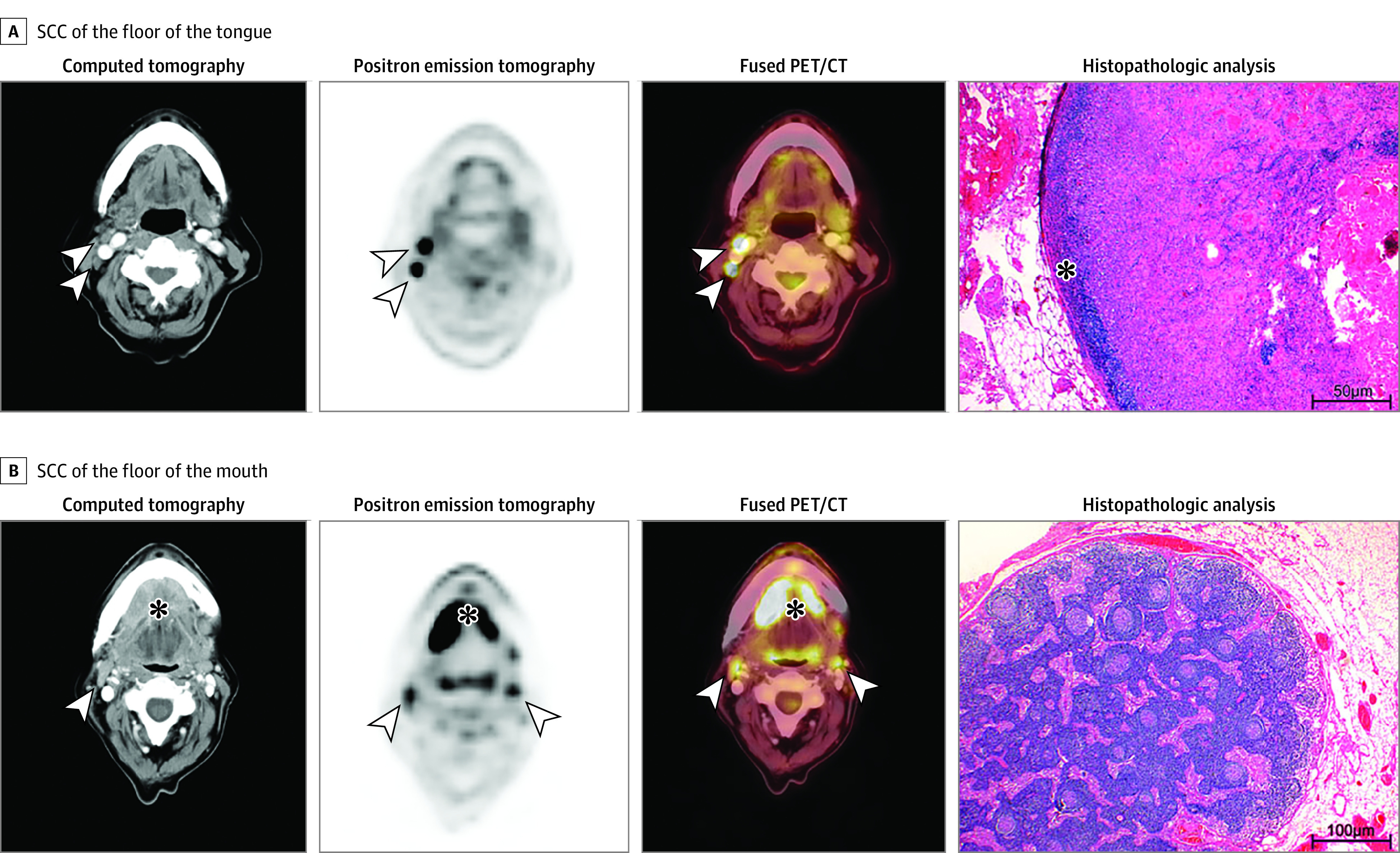
Transaxial Computed Tomographic (CT), Positron Emission Tomographic (PET), and Fused PET/CT Images A, Images of a woman aged 71 years with squamous cell carcinoma (SCC) of the floor of the tongue. Although the CT image is rather unremarkable, PET depicted intense focal tracer uptake in 2 cervical lymph nodes (arrowheads), which is highly consistent with metastatic disease. PET findings could be confirmed by histopathologic analysis with the presence of a keratinizing, moderately to poorly differentiated SCC. Only a small rim of retained lymph node tissue could be identified (star), and most of the lymph node was occupied by carcinoma. B, Images of a woman aged 59 years with SCC of the floor of the mouth (star). Both the CT and PET images depicted bilateral, enlarged/hypermetabolic lymph nodes that are suspicious for nodal metastases (arrowheads). However, histopathologic analysis revealed only inflammatory and reactive changes with prominent reactive secondary follicles of the lymph node. There was no evidence of cancer. Hemotoxylin-eosin, original magnification ×20 in histopathologic analysis.

A level-based evaluation of nodal involvement (≥1 lymph node metastasis) correctly identified 1160 of 1226 (94.6%) histopathologically assessed levels as being either positive or negative for lymph node metastases, resulting in a sensitivity of 61.5% (95% CI, 48.3%-74.8%), specificity of 96.1% (95% CI, 95.0%-97.2%), PPV of 41.0% (95% CI, 30.1%-51.9%), and NPV of 98.3% (95% CI, 97.5%-99.0%) (Table 3).

The comparison of diagnostic measures with MRI and stand-alone CT imaging showed higher values for sensitivity, specificity, PPV, and NPV of FDG PET/CT throughout all diagnostic possibilities. However, the sensitivity of FDG PET/CT (98.4%; 95% CI, 96.2%-1.00%) was significantly superior to that of MRI (69.6%; 95% CI, 61.5%-77.7%) and CT (38.4%; 95% CI, 29.9%-46.9%) only in the detection of the primary tumor. For the detection of cervical lymph node metastases, FDG PET/CT showed significantly higher specificity (83.5%; 95% CI, 75.9%-91.1%) than MRI (62.6%; 95% CI, 52.7%-72.6%), and CT (67.0%; 95% CI, 57.4%-76.7%), as well as greater PPV (FDG PET/CT: 65.1%; 95% CI, 50.9%-79.4%; MRI: 41.4%; 95% CI, 28.7%-54.1%; CT: 43.4%; 95% CI, 30.1%-56.7%) and NPV (FDG PET/CT: 92.7%; 95% CI, 87.0%-98.3%; MRI: 85.1%; 95% CI, 76.5%-93.6%; CT: 84.7%; 95% CI, 76.4%-93.0%) (Table 4; eTable 3 in the Supplement). No adverse events occurred during FDG PET/CT imaging.

**Table 4.** Comparison of Diagnostic Accuracy of FDG PET/CT, MRI, and CT in 125 Patients of Study Cohort

Compared modalities	%			
	Sensitivity	Specificity	Positive predictive value	Negative predictive value
**Primary tumor**				
FDG PET/CT vs MRI	98.4 vs 69.6^a^	NA	NA	NA
FDG PET/CT vs CT	98.4 vs 38.4^a^	NA	NA	NA
**Osseous infiltration**				
FDG PET/CT vs MRI	86.7 vs 73.3	91.6 vs 90.5	76.5 vs 71.0	95.6 vs 91.5
FDG PET/CT vs CT	86.7 vs 69.0^b^	91.6 vs 90.5	76.5 vs 69.0	95.6 vs 89.6^b^
**Lymph node metastases**				
FDG PET/CT vs MRI	82.4 vs 70.6	83.5 vs 62.6^a^	65.1 vs 41.4^a^	92.7 vs 85.1^b^
FDG PET/CT vs CT	82.4 vs 67.6	83.5 vs 67.0^c^	65.1 vs 43.4^a^	92.7 vs 84.7^b^
**Lymph node metastases in cervical sides**				
FDG PET/CT vs MRI	73.6 vs 63.1	89.1 vs 74.3^a^	59.6 vs 34.8^a^	94.0 vs 90.3^b^
FDG PET/CT vs CT	73.6 vs 57.9	89.1 vs 76.6^a^	59.6 vs 34.9^a^	94.0 vs 89.3^b^
**Lymph node metastases in cervical lymph node levels**				
FDG PET/CT vs MRI	58.3 vs 45.8	95.8 vs 92.2^a^	37.8 vs 20.6^a^	98.1 vs 97.5^b^
FDG PET/CT vs CT	58.3 vs 39.6^b^	95.8 vs 91.8^a^	37.8 vs 17.6^a^	98.1 vs 97.2^b^

Abbreviations: CT, computed tomography; FDG, 18-F fluorodeoxyglucose; NA, not available; PET, positron emission tomography.

^a^ *P* < .001.

^b^ *P* < .05.

^c^ *P* < .01.

## Discussion

In this cohort of 135 patients with newly diagnosed SCC of the oral cavity, FDG PET/CT was able to detect the primary tumor in 97.8% of the cases, thereby confirming the high sensitivity of FDG PET/CT and its superiority to stand-alone CT and MRI.^34^ All nondetected primary tumors occurred in patients with category T1 cancer with a primary tumor extension of less than or equal to 6 mm—a size that lies at the limit of PET resolution.^35^

The presence of cervical lymph node metastases is one of the most important prognostic factors in SCC of the oral cavity,^5,6,7,8^ and the accurate staging of nodal involvement is necessary. In this cohort, local lymph node metastases were present in 26.7% of the patients, with increasing frequency in higher T categories.^36^ Regarding the detection of locoregional metastases, FDG PET/CT demonstrated good sensitivity (83.3%) and specificity (84.8%), as well as high NPV (93.3%), all of which lay within the range of previous reports^24,27,28^ and were higher than the diagnostic measures of CT and MRI. Regarding side discrimination of locoregional metastasis, FDG PET/CT showed high sensitivity (73.2%) and specificity (89.9%) in this cohort. All false-negative findings, which were present in 11 (8.1%) of 135 patients, measured less than or equal to 7 mm in lymph node diameter and were predominantly present on the ipsilateral side of the tumor. In subgroup analysis for unilateral primary tumors, FDG PET/CT indicated true-negative lymph node status for the contralateral side in 97.3% of all cases.

False-positive findings were less frequent on the contralateral side and were likely caused by reactive processes in the oral cavity that are related to either ulcerating tumor growth, local tumor infiltration, or additional acute or chronic inflammatory disease. The difficulty in differentiating inflammatory processes from tumor metabolism with FDG PET/CT is well known,^37^ although tracer uptake was significantly higher for true-positive than for false-positive ratings in our study.

Level-based evaluation of the neck might help to optimize preoperative planning. Furthermore, additional information on possible skip metastases can alter the regimen of performed neck dissection.^36^ In our study, 94.6% of histopathologically assessed levels were correctly identified as being either positive or negative for lymph node metastases.

This study also expands on earlier findings on the high NPV of FDG PET/CT^28^; however, those findings were derived from a preselected cohort with head and neck SCC and clinically N0 necks after MRI, whereas we investigated a homogeneous cohort of patients with SCC of the oral cavity without the exclusion of necks with lymph node involvement after imaging with MRI. This procedure enables a better estimation of diagnostic measures and a direct comparison with MRI and stand-alone diagnostic, contrast-enhanced CT. Our results suggest that FDG PET/CT imaging is superior (Table 4, Figure, B) and that its clinical preoperative use in assessing cervical lymph node metastases could increase specificity, PPV, and NPV. The high and superior NPV for the respective cervical sides (94.0%) and levels (98.3%) should be considered before surgery to optimize neck dissection, which is important because the dissection of lymph node metastases leads to an increase in the likelihood of survival,^38^ whereas elective neck dissection is accompanied by poorer health-related quality of life.^20,39,40,41^ A more reliable estimation of lymphogenic metastases thus results in increased quality of life.

Although separate analyses of unilateral tongue carcinoma have shown poorer results than at other primary sites for ipsilateral lymph node involvement (sensitivity: 60.0%; specificity: 81.0%), correct estimation of the nodal stage on the contralateral side was possible in all 23 patients (sensitivity: 100.0%; specificity: 100.0%). Because only 1 patient had contralateral lymph node metastases, these promising results must be critically reviewed. Further investigations with a larger group size are needed for a more conclusive statement.

An additional advantage of whole-body FDG PET/CT over cervical CT and MRI is its reliable screening results for distant metastases or secondary cancer, both of which result in a necessary change to the therapeutic regimen.^9^ In this cohort, none of the patients showed distant metastasis, but a solid second primary tumor or malignant precursor lesion was confirmed in 8 of 9 cases with suspicious image findings. Whole-body FDG PET/CT might either enable early detection at a precancerous/curable stage or prevent surgical procedures for SCC of the oral cavity in the presence of an unknown advanced second primary tumor.

### Limitations

This study had limitations. Considering that metastasis could be proven in 26.7% of the patients, 73.3% of the study participants experienced a risk of overtreatment. However, because all patients underwent elective neck dissection irrespective of imaging findings, this study does not reveal the actual value of FDG PET/CT in patients who, for example, have received a PET-guided elective neck dissection without removing the contralateral cervical lymph nodes.^42^ Further prospective studies are needed to directly evaluate the influence of whole-body FDG PET/CT on patient management and follow-up.

## Conclusions

The results of this study suggest that combined FDG PET/CT is a valuable diagnostic tool in the preoperative staging of SCC of the oral cavity. Use of FDG PET/CT was associated with a high NPV and was superior to stand-alone morphologic imaging.

## Notes

Supplement. **eMethods.** Detailed Methods **eReferences** **eTable 1.** Estimated and True Conditions Subgroup Analysis **eTable 2.** Diagnostic Accuracy Subgroup Analysis **eTable 3.** Diagnostic Accuracy of FDG PET/CT, MRI and CT for Oral Cavity Squamous Cell Carcinoma and Nodal Metastatic Involvement in 125 Patients of the Study Cohort **eFigure.** Study Design and Patient Inclusion

## References

[1] Hernández-Guerrero JC, Jacinto-Alemán LF, Jiménez-Farfán MD, Macario-Hernández A, Hernández-Flores F, Alcántara-Vázquez A. Prevalence trends of oral squamous cell carcinoma: Mexico City’s General Hospital experience. Med Oral Patol Oral Cir Bucal. 2013;18(2):e306-e311. doi:10.4317/medoral.18043 23385493 PMC3613885

[2] Sadick M, Schoenberg SO, Hoermann K, Sadick H. Current oncologic concepts and emerging techniques for imaging of head and neck squamous cell cancer. GMS Curr Top Otorhinolaryngol Head Neck Surg. 2012;11:Doc08. doi:10.3205/cto000090 23320060 PMC3544205

[3] Bagan J, Sarrion G, Jimenez Y. Oral cancer: clinical features. Oral Oncol. 2010;46(6):414-417. doi:10.1016/j.oraloncology.2010.03.009 20400366

[4] Tandon P, Dadhich A, Saluja H, Bawane S, Sachdeva S. The prevalence of squamous cell carcinoma in different sites of oral cavity at our Rural Health Care Centre in Loni, Maharashtra—a retrospective 10-year study. Contemp Oncol (Pozn). 2017;21(2):178-183. doi:10.5114/wo.2017.68628 28947890 PMC5611509

[5] Amit M, Yen TC, Liao CT, ; International Consortium for Outcome Research (ICOR) in Head and Neck Cancer. Improvement in survival of patients with oral cavity squamous cell carcinoma: an international collaborative study. Cancer. 2013;119(24):4242-4248. doi:10.1002/cncr.28357 24114787

[6] van Dijk BA, Brands MT, Geurts SM, Merkx MA, Roodenburg JL. Trends in oral cavity cancer incidence, mortality, survival and treatment in the Netherlands. Int J Cancer. 2016;139(3):574-583. doi:10.1002/ijc.30107 27038013

[7] DiTroia JF. Nodal metastases and prognosis in carcinoma of the oral cavity. Otolaryngol Clin North Am. 1972;5(2):333-342. doi:10.1016/S0030-6665(20)32999-6 4557048

[8] Kumar T, Patel MD. Pattern of lymphatic metastasis in relation to the depth of tumor in oral tongue cancers: a clinico pathological correlation. Indian J Otolaryngol Head Neck Surg. 2013;65(suppl 1):59-63. doi:10.1007/s12070-012-0504-y 24427617 PMC3718938

[9] Sparano A, Weinstein G, Chalian A, Yodul M, Weber R. Multivariate predictors of occult neck metastasis in early oral tongue cancer. Otolaryngol Head Neck Surg. 2004;131(4):472-476. doi:10.1016/j.otohns.2004.04.008 15467620

[10] Teichgraeber JF, Clairmont AA. The incidence of occult metastases for cancer of the oral tongue and floor of the mouth: treatment rationale. Head Neck Surg. 1984;7(1):15-21. doi:10.1002/hed.2890070105 6490380

[11] Haigentz M Jr, Hartl DM, Silver CE, . Distant metastases from head and neck squamous cell carcinoma— part III: treatment. Oral Oncol. 2012;48(9):787-793. doi:10.1016/j.oraloncology.2012.03.019 22516376

[12] de Bree R, Castelijns JA, Hoekstra OS, Leemans CR. Advances in imaging in the work-up of head and neck cancer patients. Oral Oncol. 2009;45(11):930-935. doi:10.1016/j.oraloncology.2009.07.011 19692289

[13] Edge SB, Compton CC. The American Joint Committee on Cancer: the 7th edition of the AJCC cancer staging manual and the future of TNM. Ann Surg Oncol. 2010;17(6):1471-1474. doi:10.1245/s10434-010-0985-4 20180029

[14] Nahmias C, Carlson ER, Duncan LD, . Positron emission tomography/computerized tomography (PET/CT) scanning for preoperative staging of patients with oral/head and neck cancer. J Oral Maxillofac Surg. 2007;65(12):2524-2535. doi:10.1016/j.joms.2007.03.010 18022480

[15] Rumboldt Z, Day TA, Michel M. Imaging of oral cavity cancer. Oral Oncol. 2006;42(9):854-865. doi:10.1016/j.oraloncology.2006.01.010 16798060

[16] Fan S, Tang QL, Lin YJ, . A review of clinical and histological parameters associated with contralateral neck metastases in oral squamous cell carcinoma. Int J Oral Sci. 2011;3(4):180-191. doi:10.4248/IJOS11068 22010576 PMC3469975

[17] Lea J, Bachar G, Sawka AM, . Metastases to level IIb in squamous cell carcinoma of the oral cavity: a systematic review and meta-analysis. Head Neck. 2010;32(2):184-190. doi:10.1002/hed.21163 19626638

[18] Noguti J, De Moura CF, De Jesus GP, . Metastasis from oral cancer: an overview. Cancer Genomics Proteomics. 2012;9(5):329-335. 22990112

[19] Wolff KD, Follmann M, Nast A. The diagnosis and treatment of oral cavity cancer. Dtsch Arztebl Int. 2012;109(48):829-835. doi:10.3238/arztebl.2012.0829 23248713 PMC3523261

[20] Spalthoff S, Zimmerer R, Jehn P, Gellrich NC, Handschel J, Krüskemper G. Neck dissection’s burden on the patient: functional and psychosocial aspects in 1,652 patients with oral squamous cell carcinomas. J Oral Maxillofac Surg. 2017;75(4):839-849. doi:10.1016/j.joms.2016.09.037 27776222

[21] Cho JK, Ow TJ, Lee AY, . Preoperative ^18^F-FDG-PET/CT vs contrast-enhanced CT to identify regional nodal metastasis among patients with head and neck squamous cell carcinoma. Otolaryngol Head Neck Surg. 2017;157(3):439-447. doi:10.1177/0194599817703927 28608737 PMC6372372

[22] Dammann F, Horger M, Mueller-Berg M, . Rational diagnosis of squamous cell carcinoma of the head and neck region: comparative evaluation of CT, MRI, and ^18^FDG PET. AJR Am J Roentgenol. 2005;184(4):1326-1331. doi:10.2214/ajr.184.4.01841326 15788619

[23] Hirshoren N, Olayos E, Herschtal A, Ravi Kumar AS, Gyorki DE. Preoperative positron emission tomography for node-positive head and neck cutaneous squamous cell carcinoma. Otolaryngol Head Neck Surg. 2018;158(1):122-126. doi:10.1177/0194599817731735 28925330

[24] Krabbe CA, Balink H, Roodenburg JL, Dol J, de Visscher JG. Performance of 18F-FDG PET/contrast-enhanced CT in the staging of squamous cell carcinoma of the oral cavity and oropharynx. Int J Oral Maxillofac Surg. 2011;40(11):1263-1270. doi:10.1016/j.ijom.2011.06.023 21824748

[25] Subramaniam RM, Agarwal A, Colucci A, Ferraro R, Paidpally V, Mercier G. Impact of concurrent diagnostic level CT with PET/CT on the utilization of stand-alone CT and MRI in the management of head and neck cancer patients. Clin Nucl Med. 2013;38(10):790-794. doi:10.1097/RLU.0b013e31829f8ca5 23917783

[26] Wallowy P, Diener J, Grünwald F, Kovács AF. 18F-FDG PET for detecting metastases and synchronous primary malignancies in patients with oral and oropharyngeal cancer. Nuklearmedizin. 2009;48(5):192-199. doi:10.3413/nukmed-0242 19623408

[27] Pentenero M, Cistaro A, Brusa M, . Accuracy of 18F-FDG-PET/CT for staging of oral squamous cell carcinoma. Head Neck. 2008;30(11):1488-1496. doi:10.1002/hed.20906 18767178

[28] Lowe VJ, Duan F, Subramaniam RM, . Multicenter trial of [^18^F]fluorodeoxyglucose positron emission tomography/computed tomography staging of head and neck cancer and negative predictive value and surgical impact in the N0 Neck: results from ACRIN 6685. J Clin Oncol. 2019;37(20):1704-1712. doi:10.1200/JCO.18.01182 30768363 PMC6638599

[29] Bossuyt PM, Reitsma JB, Bruns DE, ; STARD Group. STARD 2015: an updated list of essential items for reporting diagnostic accuracy studies. Radiology. 2015;277(3):826-832. doi:10.1148/radiol.2015151516 26509226

[30] Forghani R, Yu E, Levental M, Som PM, Curtin HD. Imaging evaluation of lymphadenopathy and patterns of lymph node spread in head and neck cancer. Expert Rev Anticancer Ther. 2015;15(2):207-224. doi:10.1586/14737140.2015.978862 25385488

[31] Som PM, Curtin HD, Mancuso AA. Imaging-based nodal classification for evaluation of neck metastatic adenopathy. AJR Am J Roentgenol. 2000;174(3):837-844. doi:10.2214/ajr.174.3.1740837 10701636

[32] Robbins KT, Clayman G, Levine PA, ; American Head and Neck Society; American Academy of Otolaryngology–Head and Neck Surgery. Neck dissection classification update: revisions proposed by the American Head and Neck Society and the American Academy of Otolaryngology–Head and Neck Surgery. Arch Otolaryngol Head Neck Surg. 2002;128(7):751-758. doi:10.1001/archotol.128.7.751 12117328

[33] Ng SH, Yen TC, Chang JT, . Prospective study of [18F]fluorodeoxyglucose positron emission tomography and computed tomography and magnetic resonance imaging in oral cavity squamous cell carcinoma with palpably negative neck. J Clin Oncol. 2006;24(27):4371-4376. doi:10.1200/JCO.2006.05.7349 16983105

[34] Roh JL, Yeo NK, Kim JS, . Utility of 2-[18F] fluoro-2-deoxy-D-glucose positron emission tomography and positron emission tomography/computed tomography imaging in the preoperative staging of head and neck squamous cell carcinoma. Oral Oncol. 2007;43(9):887-893. doi:10.1016/j.oraloncology.2006.10.011 17207656

[35] Karlberg AM, Sæther O, Eikenes L, Goa PE. Quantitative comparison of PET performance–Siemens Biograph mCT and mMR. EJNMMI Phys. 2016;3(1):5. doi:10.1186/s40658-016-0142-7 26911722 PMC4766138

[36] Coskun HH, Medina JE, Robbins KT, . Current philosophy in the surgical management of neck metastases for head and neck squamous cell carcinoma. Head Neck. 2015;37(6):915-926. doi:10.1002/hed.23689 24623715 PMC4991629

[37] Wong RJ. Current status of FDG-PET for head and neck cancer. J Surg Oncol. 2008;97(8):649-652. doi:10.1002/jso.21018 18493944

[38] D’Cruz AK, Vaish R, Kapre N, ; Head and Neck Disease Management Group. Elective versus therapeutic neck dissection in node-negative oral cancer. N Engl J Med. 2015;373(6):521-529. doi:10.1056/NEJMoa1506007 26027881

[39] de Melo GM, Ribeiro KC, Kowalski LP, Deheinzelin D. Risk factors for postoperative complications in oral cancer and their prognostic implications. Arch Otolaryngol Head Neck Surg. 2001;127(7):828-833. 11448358

[40] McDonald C, Lowe D, Bekiroglu F, Schache A, Shaw R, Rogers SN. Health-related quality of life in patients with T1N0 oral squamous cell carcinoma: selective neck dissection compared with wait and watch surveillance. Br J Oral Maxillofac Surg. 2019;57(7):649-654. doi:10.1016/j.bjoms.2019.05.021 31230853

[41] Peters TT, van Dijk BA, Roodenburg JL, van der Laan BF, Halmos GB. Relation between age, comorbidity, and complications in patients undergoing major surgery for head and neck cancer. Ann Surg Oncol. 2014;21(3):963-970. doi:10.1245/s10434-013-3375-x 24248531

[42] Mehanna H, Wong WL, McConkey CC, ; PET-NECK Trial Management Group. PET-CT surveillance versus neck dissection in advanced head and neck cancer. N Engl J Med. 2016;374(15):1444-1454. doi:10.1056/NEJMoa1514493 27007578

[43] The R Foundation. The R Project for Statistical Computing. Accessed March 31, 2021. http://www.R-project.org/

