# Supplementary material for: Accuracy of 18-F Fluorodeoxyglucose Positron Emission Tomographic/Computed Tomographic Imaging in Primary Staging of Squamous Cell Carcinoma of the Oral Cavity
Source: JAMA Netw Open. 2021 Apr 21;4(4):e217083. doi: 10.1001/jamanetworkopen.2021.7083 (PMC8060833; doi:10.1001/jamanetworkopen.2021.7083)
Supplement: Supplement. — eMethods. Detailed Methods eReferences eTable 1. Estimated and True Conditions Subgroup Analysis eTable 2. Diagnostic Accuracy Subgroup Analysis eTable 3. Diagnostic Accuracy of FDG PET/CT, MRI and CT for Oral Cavity Squamous Cell Carcinoma and Nodal Metastatic Involvement in 125 Patients of the Study Cohort eFigure. Study Design and Patient Inclusion [file jamanetwopen-e217083-s001.pdf]

Supplementary Online Content

Linz C, Brands RC, Herterich T, et al. Accuracy of 18-F fluorodeoxyglucose positron emission tomographic/computed tomographic imaging in primary staging of squamous cell carcinoma of the oral cavity. *JAMA Netw Open*. 2021;4(4):e217083. doi:10.1001/jamanetworkopen.2021.7083

eMethods. Detailed Methods

eReferences

eTable 1. Estimated and True Conditions Subgroup Analysis

eTable 2. Diagnostic Accuracy Subgroup Analysis

eTable 3. Diagnostic Accuracy of FDG PET/CT, MRI and CT for Oral Cavity Squamous Cell Carcinoma and Nodal Metastatic Involvement in 125 Patients of the Study Cohort

eFigure. Study Design and Patient Inclusion

This supplementary material has been provided by the authors to give readers additional information about their work.

© 2021 Linz C et al. *JAMA Network Open.*

eMethods. Detailed Methods

18F-FDG PET/CT imaging

Imaging for all patients was performed on an integrated PET/CT scanner (Siemens Biograph mCT 64, Siemens Healthineers, Knoxville, USA). Prior to imaging, patients fasted for at least 4–6 h. Blood glucose levels were measured and confirmed to be below 160 mg/dl before the intravenous injection of 300 ± 25 MBq 18F-FDG. After a distribution period of 60 min, PET emission data were acquired in 3D-mode with a 200 × 200 matrix with 2-min emission time per bed position from the vertex of the skull to the proximal thighs. Consecutively, transmission data were acquired using contrast-enhanced spiral CT (dose modulation with a quality reference of 180 mAs, 120 kV, a 512 × 512 matrix, 5-mm slice thickness, an increment of 30 mm/s, a rotation time of 0.5 s, and a pitch index of 1.4). Furthermore, a dedicated acquisition of the head and neck with one bed position, a 3-min emission time, and a contrast-enhanced CT was performed (180 mAs, 120 kV, a 512 × 512 matrix, 3-mm slice thickness, an increment of 30 mm/s, a rotation time of 1.0 s, and a pitch index of 0.9). PET data were reconstructed iteratively (3 iterations, 24 subsets, a Gaussian filtering of 2.0 mm full width at half maximum) with attenuation correction using dedicated standard software (HD. PET, Siemens Esoft, Siemens Healthineers, Erlangen, Germany).

MRI imaging

MRI scans focused on the head and neck area of the patients. All MRI scans were acquired using a 1.5T scanner (Siemens Magnetom Avanto fit, Siemens Healthcare, Erlangen, Germany) (n = 95) or a 3.0T scanner (Siemens Magnetom Prisma or Skyra, Siemens Healthcare, Erlangen, Germany) (n = 30) with a 64-channel head/neck coil for signal reception. Standard in-house MRI sequences included: coronal T2 weighted inversion recovery magnitude (TIRM), axial T2 weighted imaging, coronal T1 weighted imaging without and with contrast enhancement including a coronal substraction, axial

© 2021 Linz C et al. *JAMA Network Open.*

T1 weighted imaging with fat saturation and with contrast enhancement and axial diffusion weighted imaging. 14 patients were scanned before November 2013 when standard sequences were changed. Scans prior to the protocol change included a sagittal T1 weighted imaging with fat saturation and with contrast enhancement instead of an axial T1 weighted imaging with fat saturation and with contrast enhancement.

The sequence protocols using a 1.5T scanner were as follows: T2-TIRM protocol parameters: TR/TE /TI 4950/66/140, field of view = 300 x 300 mm2, 33 slices, slice thickness = 4 mm, matrix = 384 × 384; T2-TSE protocol parameters: TR/TE = 5500/91 ms, field of view = 210 x 210 mm2, 23 slices, slice thickness = 4 mm, matrix = 384 × 384; coronal T1 protocol parameters: TR/TE = 483/12 ms, field of view = 210 x 210 mm2, 33 slices, slice thickness = 4 mm, matrix = 504 × 448; T1 TSE FS DIXON protocol parameters: TR/TE = 610/14 ms, field of view = 210 x 210 mm2, 13 slices, slice thickness = 4 mm, matrix = 384 × 384; diffusion weighted imaging parameters TR/TE 3300/92, field of view = 260 x 245 mm2, 23 slices, slice thickness = 4 mm, matrix = 178 × 178; sagittal T1 FS DIXON: TR/TE 400/13, field of view = 300 x 300 mm2, 18 slices, slice thickness = 4 mm, matrix = 448 × 448.

The sequence protocols using a 3T scanner were as follows: T2-TIRM protocol parameters TR/TE /TI 2900/80/210, field of view = 300 x 300 mm2, 35 slices, slice thickness = 4 mm, matrix = 448 × 448; T2-TSE protocol parameters: TR/TE = 7490/113 ms, field of view = 200 x 200 mm2, 29 slices, slice thickness = 4 mm, matrix = 448 × 448; coronal T1 protocol parameters: TR/TE = 480/11 ms, field of view = 300 x 300 mm2, 35 slices, slice thickness = 4 mm, matrix = 448× 448; T1 TSE FS DIXON protocol parameters: TR/TE = 750/13 ms, field of view = 200 x 200 mm2, 21 slices, slice thickness = 4 mm, matrix = 384 × 384; diffusion weighted imaging parameters TR/TE 6400/81, field of view = 260 x 211 mm2, 29 slices, slice thickness = 4 mm, matrix = 200 × 162; sagittal T1 FS DIXON: TR/TE 535/11, field of

© 2021 Linz C et al. *JAMA Network Open.*

view = 300 x 300 mm2, 28 slices, slice thickness = 5 mm, matrix = 312× 512. Gadoterat-acid (Dotagraf; Jenapharm GmbH & Co. KG, Jena, Germany) was administered to the patients (dose, 0.1 mmol/kg) followed by a 20-ml saline flush via a power injector (Spectris MR Injector; Medrad) at a rate of 1 ml/second.

18F-FDG PET/CT image analysis

Two experienced, board-certified nuclear medicine physicians (J.B. and C.L.) independently rated whole-body- and cervical PET/CT on a syngo.via workstation (Siemens Healthineers, Erlangen, Germany). The foci of increased tracer uptake with reference to normal tissue and blood pool and/or to the presence of morphological alterations on CT images were recorded as being positive for tumor involvement. The localization, expansion, and infiltration of osseous structures as well as the presence and number of nodal metastases were recorded for each cervical lymph node level. Lymph node levels were assessed according to the imaging-based nodal classification [*1, 2*]. Furthermore, whole-body scans were evaluated for distant nodal and organ metastasis. Any initial difference in rating between the two readers was resolved via a subsequent consensus reading. If present, the maximal (SUV_{max}) and peak standardized uptake values (SUV_{peak}) of the primary tumor, the hottest cervical lymph node, and the hottest distant metastasis were measured.

MR image analysis

All MRI scans were rated on a MERLIN Diagnostic Workcenter (Phönix-PACS Gmbh, Freiburg, Germany) by one experienced, board-certified radiologist (A.D.) who had access to all clinical data but was blinded to [^{18}F]FDG-PET/CT results. MRI scans were analyzed according to previous published methods [*3, 4*]. The localization, expansion, and infiltration of osseous structures as well as the presence and number of nodal metastasis were recorded for each cervical lymph node level. Lymph node levels were assessed according to the imaging-based nodal classification [*1, 2*] .

© 2021 Linz C et al. *JAMA Network Open.*

CT image analysis

Contrast-enhanced cervical CT from combined [^{18}F]FDG-PET/CT acquisition served as simulation of standalone CT. One experienced, board-certified radiologists (A.D.) rated independently cervical CT scans and whole body CT scans on a MERLIN Diagnostic Workcenter (Phönix-PACS Gmbh, Freiburg, Germany). He had access to all clinical data but was blinded to [^{18}F]FDG-PET/CT and MR results.

Morphological alterations on CT images were recorded as being positive for tumor involvement. The localization, expansion, and infiltration of osseous structures as well as the presence and number of nodal metastasis were recorded for each cervical lymph node level. Lymph nodes with short-axis diameter in axial plane ≥10 mm were rated positive. Furthermore, cluster of three or more borderline nodes, necrotic/cystic areas and evidence of extra-nodal extension were rated as signs of malignancy [*5*-*7*]. Lymph node levels were assessed according to the imaging-based nodal classification [*1, 2*].

The diagnostic performances of (i) 18F-FDG PET/CT, (ii) MRI and (iii) CT in 125 patients are listed in eTable 2. The comparison between the methods is listed in Table 3 of the article.

© 2021 Linz C et al. *JAMA Network Open.*

eReferences

1. Campbell, R.S., Baker, E., Chippindale, A.J., Wilson, G., McLean, N., Soames, J.V., and Reed, M.F., *MRI T staging of squamous cell carcinoma of the oral* 1. Forghani, R., Yu, E., Levental, M., Som, P.M., and Curtin, H.D., Imaging evaluation of lymphadenopathy and patterns of lymph node spread in head and neck cancer. *Expert Rev Anticancer Ther*, **2015**. 15(2): 207-24.
2. Som, P.M., Curtin, H.D., and Mancuso, A.A., Imaging-based nodal classification for evaluation of neck metastatic adenopathy. *AJR Am J Roentgenol*, **2000**. 174(3): 837-44.
3. Campbell, R.S., Baker, E., Chippindale, A.J., et al., MRI T staging of squamous cell carcinoma of the oral cavity: radiological-pathological correlation. *Clin Radiol*, **1995**. 50(8): 533-40.
4. Trotta, B.M., Pease, C.S., Rasamny, J.J., Raghavan, P., and Mukherjee, S., Oral cavity and oropharyngeal squamous cell cancer: key imaging findings for staging and treatment planning. *Radiographics*, **2011**. 31(2): 339-54.
5. Chung, M.S., Choi, Y.J., Kim, S.O., et al., A Scoring System for Prediction of Cervical Lymph Node Metastasis in Patients with Head and Neck Squamous Cell Carcinoma. *AJNR Am J Neuroradiol*, **2019**. 40(6): 1049-1054.
6. Lan, M., Huang, Y., Chen, C.Y., et al., Prognostic Value of Cervical Nodal Necrosis in Nasopharyngeal Carcinoma: Analysis of 1800 Patients with Positive Cervical Nodal Metastasis at MR Imaging. *Radiology*, **2015**. 276(2): 536-44.
7. van den Brekel, M.W., Stel, H.V., Castelijns, J.A., et al., Cervical lymph node metastasis: assessment of radiologic criteria. *Radiology*, **1990**. 177(2): 379-84.

© 2021 Linz C et al. *JAMA Network Open.*

eTable 1. Estimated and True Conditions Subgroup Analysis

**Subgroup analysis for lymph node metastases according to primary site localization**						
		No.				
		Total	True-positive	False-positive	False-negative	True-negative
Primary localization	Neck dissection					
Primary of oral cavity without tongue						
Central	Both sides	40	4	4	3	29
Lateral	Ipsilateral	80	16	10	2	52
Lateral	Contralateral	55	3	1	2	49
Primary of tongue						
Lateral	Ipsilateral	31	6	4	4	17
Lateral	Contralateral	23	1	0	0	22
All primary sites						
Lateral	Ipsilateral	111	22	14	6	69
Lateral	Contralateral	78	4	1	2	71

Central indicates central or bilateral; lateral, left or righ side.

© 2021 Linz C et al. *JAMA Network Open.*

eTable 2. Diagnostic Accuracy Subgroup Analysis

Subgroup analysis for lymph node metastases according to primary site localization						
		% (95% CI)				
		Prevalence	Sensitivity	Specificity	Positive predictive value	Negative predictive value
Primary localization	Neck dissection					
Primary of oral cavity without tongue						
Central	Both sides	17.5 (5.7-29.3)	57.1 (20.5-93.8)	87.9 (76.7-99.0)	50.0 (15.4-84.6)	90.6 (80.5-100.0)
Lateral	Ipsilateral	22.5 (13.3-31.7)	88.9 (74.4-100.0)	83.9 (74.7-93.0)	61.5 (42.8-80.2)	96.3 (91.3-100.0)
Lateral	Contralateral	9.1 (1.5-16.7)	60.0 (17.1-100.0)	98.0 (94.1-100.0)	75.0 (21.7-100.0)	96.1 (90.8-100.0)
Primary of tongue						
Lateral	Ipsilateral	32.3 (15.8-48.7)	60.0 (29.6-90.4)	81.0 (64.2-97.7)	60.0 (29.6-90.4)	81.0 (64.2-97.7)
Lateral	Contralateral	4.4 (0.0-12.7)	100.0 (100.0-100.0)	100.0 (100.0-100.0)	100.0 (100.0-100.0)	100.0 (100.0-100.0)
All primary sites						
Lateral	Ipsilateral	25.2 (17.1-33.3)	78.6 (63.4-93.8)	83.1 (75.1-91.2)	61.1 (45.2-77.0)	92.0 (85.9-98.1)
Lateral	Contralateral	7.7 (1.8-13.6)	66.7 (28.9-100.0)	98.6 (95.9-100.0)	80.0 (44.9-100.0)	97.3 (93.5-100.0)

Central indicates central or bilateral; lateral, left or righ side.

© 2021 Linz C et al. *JAMA Network Open.*

eTable 3 Diagnostic accuracy of 18F-FDG-PET/CT, MRI and CT for oral squamous cell carcinoma and nodal metastatic involvement in 125 patients of the study cohort

Method	Total (No.)	TP (No.)	FP (No.)	FN (No.)	TN (No.)	Prevalence (%)	Sensitivity (%)	Specificity (%)	Positive predictive value (%)	Negative predictive value (%)
Primary tumor										
FDG PET/CT	125	123	0	2	0	100.0 (100.0-100.0)	98.4 (96.2-1.00)	n/a	n/a	n/a
MRI		87	0	38	0		69.6 (61.5-77.7)	n/a	n/a	n/a
CT		48	0	77	0		38.4 (29.9-46.9)	n/a	n/a	n/a
Osseous Infiltration										
FDG PET/CT	125	26	8	4	87	24.0 (16.5-31.5)	86.7 (74.5-98.8)	91.6 (86.0-97.2)	76.5 (62.2-90.7)	95.6 (91.4-99.8)
MRI		22	9	8	86		73.3 (57.5-89.2)	90.5 (84.6-96.4)	71.0 (55.0-86.9)	91.5 (85.8-97.1)
CT		20	9	10	86		69.0 (52.1-85.8)	90.5 (84.6-96.4)	69.0 (52.1-85.8)	89.6 (83.5-95.7)
Lymph node metastases										
FDG PET/CT	125	28	15	6	76	27.2 (19.4-35.0)	82.4 (69.5-95.2)	83.5 (75.9-91.1)	65.1 (50.9-79.4)	92.7 (87.0-98.3)
MRI		24	34	10	57		70.6 (55.3-85.9)	62.6 (52.7-72.6)	41.4 (28.7-54.1)	85.1 (76.5-93.6)
CT		23	30	11	61		67.6 (51.9-83.4)	67.0 (57.4-76.7)	43.4 (30.1-56.7)	84.7 (76.4-93.0)
Lymph node metastases in cervical sides										
FDG PET/CT	213	28	19	10	156	17.8 (12.7-23.0)	73.6 (59.7-87.7)	89.1 (84.5-93.8)	59.6 (45.5-73.6)	94.0 (90.4-97.6)
MRI		24	45	14	130		63.1 (47.8-78.5)	74.3 (67.8-80.8)	34.8 (23.5-46.0)	90.3 (85.4-95.1)
CT		22	41	16	134		57.9 (42.2-73.6)	76.6 (70.3-82.8)	34.9 (23.1-46.7)	89.3 (84.4-94.3)
Lymph node metastases in cervical lymph node levels										
FDG PET/CT	1139	28	46	20	1045	4.2 (3.1-5.4)	58.3 (44.4-72.3)	95.8 (94.6-97.0)	37.8 (26.8-48.9)	98.1 (97.3-98.9)
MRI		22	85	26	1006		45.8 (31.7-59.9)	92.2 (90.6-93.8)	20.6 (12.9-28.2)	97.5 (96.5-98.4)
CT		19	89	29	1002		39.6 (25.7-53.4)	91.8 (90.2-93.5)	17.6 (10.4-24.8)	97.2 (96.2-98.2)

Abbreviations: TP = true positive; FP = false positive; FN = false positive; TN = true negative; n/a = not applicable.

© 2021 Linz C et al. *JAMA Network Open.*

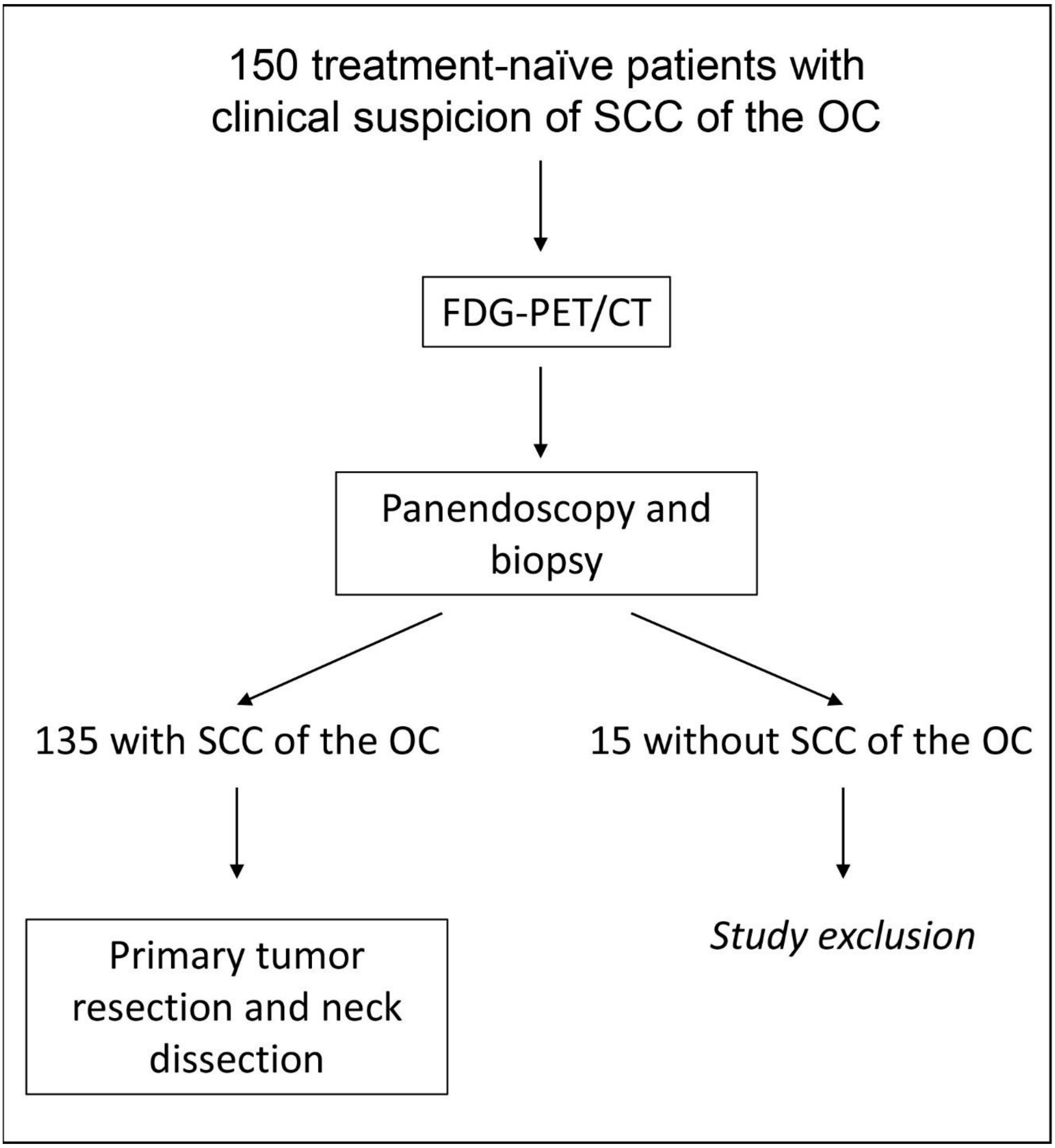

eFigure. Study design and patient inclusion. SCC = squamous cell carcinoma, OC = oral cavity.

© 2021 Linz C et al. *JAMA Network Open.*
